# Prevalence of pain in women living with HIV aged 45–60: associated factors and impact on patient-reported outcomes

**DOI:** 10.1080/09540121.2021.1887445

**Published:** 2021-02-22

**Authors:** Caroline A. Sabin, Hajra Okhai, Rageshri Dhairyawan, Katharina Haag, Fiona Burns, Richard Gilson, Lorraine Sherr, Shema Tariq

**Affiliations:** a Institute for Global Health, UCL, London, UK; b Department of Infection and Immunity, Barts Health NHS Trust, London, UK; c Royal Free London NHS Foundation Trust, London, UK

**Keywords:** HIV, menopause, pain, predictors, insomnia, depression

## Abstract

As the population of women with HIV ages, an increasing proportion are experiencing the menopause, with potential associated pain. Among 844 participants in the Positive Transitions Through the Menopause (PRIME) study (72.3% black African; median age 49 (interquartile-range 47–53) years; 20.9%, 44.0% and 35.1% pre-, peri- and post-menopausal), 376 (44.6%) and 73 (8.7%) reported moderate or extreme pain. Women had been diagnosed with HIV for 14 (9–18) years, 97.7% were receiving antiretroviral therapy and 88.4% had a suppressed viral load. In adjusted ordinal logistic regression, peri-menopausal status (adjusted odds ratio (1.80) [95% confidence interval 1.22–2.67]), current smoking (1.85 [1.11–3.09]), number of comorbid conditions (1.95 [1.64–2.33] /condition) and longer duration of HIV (1.12 [1.00–1.24]/5 years) were independently associated with increased reported pain, whereas being in full-time work (0.61 [0.45–0.83]) and having enough money for basic needs (0.47 [0.34–0.64]) were associated with decreased pain reporting. Increasing pain was independently related to insomnia symptoms (moderate: 2.76 [1.96–3.90]; extreme: 8.09 [4.03–16.24]) and severe depressive symptoms (PHQ4 ≥ 6; moderate: 3.96 [2.50–6.28]; extreme: 9.13 [4.45–18.72]). Whilst our analyses cannot determine the direction of any associations, our findings point to the importance of eliciting a history of pain and addressing symptoms in order to improve wellbeing.

## Introduction

The widespread roll-out of antiretroviral therapy (ART) for HIV infection has resulted in increased life expectancies for those receiving ART in many settings (Antiretroviral Therapy Cohort Collaboration, 2017; May et al., 2014). As a result, the population of people with HIV is ageing, bringing with it an increased spectrum of co-morbidities and health concerns. Widespread pain is commonly reported by people with HIV (da Silva et al., 2017; Merlin et al., 2012; Parker et al., 2014; Sabin et al., 2018) and negatively impacts on many health outcomes, including quality-of-life and mental health (da Silva et al., 2017; Sabin et al., 2018; Scott et al., 2018). Pain may also have an impact on a person’s ability to take their ART as prescribed (Lampe et al., 2010; Sherr et al., 2008) and thus has the potential to contribute to continuing HIV-related morbidity and onward HIV transmission. Quality-of-life is directly affected by pain, affecting the ability to maintain employment, and negatively impacting on enjoyment and mood (Burke et al., 2015).

Globally, around half of the world’s population of 38 million people living with HIV is female (World Health Organization, 2020); this group is also ageing. In the UK, in 2018, of the 29,712 women diagnosed with HIV who attended HIV care services, 15,576 were aged 35–49 years and 8643 aged 50–64 years (Public Health England, 2020), groups likely to include a high proportion of women of menopausal age. Pain, most commonly muscle and joint pain, is a recognised menopausal symptom (Rindner et al., 2017). Yet, despite the known associations between pain and both HIV and the menopause, relatively little is known about the prevalence, predictors or experiences of pain in older women with HIV, or about the potential impact of pain on other aspects of women’s lives, including their mental health. One study (Schnall et al., 2018) has reported that women experience a greater burden of muscle aches and joint pain than men, with this burden being higher in post-menopausal women even after adjustment for potential confounders. A recent Canadian study found a marginal association between severe menopausal symptoms (including pain) and sub-optimal ART adherence (Duff et al., 2018). This lack of published information on the prevalence or impact of pain among women with HIV of menopausal age contributes to a lack of awareness of the importance of this symptom among those providing care for women living with HIV, and limits the development of evidence-based guidelines for the holistic management of women with HIV.

To address this evidence gap, in this paper we describe the prevalence of pain among women living with HIV aged 45–60 years in the Positive Transitions Through the Menopause (PRIME) study, one of the largest studies internationally investigating the impact of menopause in women with HIV, and identify demographic, lifestyle and clinical factors associated with pain severity in this group. We also describe the association of pain severity with severe depressive symptoms and insomnia, outcomes which are highly prevalent in people with HIV as well as being common menopausal symptoms.

## Materials and methods

### The PRIME study

The PRIME Study is a multi-centre, cross-sectional, mixed-methods observational study of the impact of the menopause transition on the health and well-being of women living with HIV. Full details of the study methods are described elsewhere (Tariq et al., 2019). In brief, women (defined as female sex at birth) aged 45–60 were recruited from one of 21 NHS HIV clinics across England between February 2016 and June 2017. Women were eligible to participate regardless of menopausal status, but were ineligible if they had experienced surgical menopause or had a history of anything that might disrupt their bleeding pattern. We excluded women whose last menstrual period was more than 60 months before (unless currently on hormone replacement therapy (HRT)) as we aimed to capture women who were most likely to be experiencing symptoms. All women gave informed consent to participate and the study received ethical approval from the South East Coast-Surrey Research Ethics Committee (REF 15/0735) on behalf of all National Health Service (NHS) sites.

The self-completed paper questionnaires comprised questions relating to demographic/social factors; prior diagnosis of one of nine comorbidities, selected due to their known association with HIV infection and/or potential impact on the use of HRT (Hepatitis B/C, hypertension, diabetes, cardiovascular disease (CVD), stroke, osteoporosis, breast cancer and depression); current lifestyle factors (smoking, harmful alcohol use [Alcohol Use Disorders Identification Test (AUDIT-C, auditscreen.org) score ≥5], recreational drug use in the previous 3 months); HIV-related history (years since HIV diagnosis, most recent clinical markers including CD4 count and HIV viral load); menopause-related symptoms (Menopause Rating Scale, MRS (ZEG Berlin, (2021))) and care-seeking; and sexual function. Information on current pain/discomfort was collected through a single question on the validated EuroQoL-5D-3L scale (EQ-5D; EuroQoL Group, 1990), in which participants were asked to classify current pain/discomfort as none, moderate or extreme.

Menopausal status was determined from self-reported menstrual pattern (without biological confirmation) (Brambilla et al., 1994), and categorised according to the modified Stages of Reproductive Aging Workshop (STRAW) + 10 criteria as pre-, peri- and post-menopausal (Harlow et al., 2012). With participants’ consent, questionnaire data were supplemented by routinely collected clinical data, including nadir and current CD4 count, baseline and current HIV viral load, and current ART regimen.

### Statistical methods

The present analysis was restricted to women with available data on pain. Demographic and lifestyle characteristics, and the prevalence of each of nine pre-specified comorbidities were compared between those with different levels of reported pain and were tested for significance using Chi-squared tests or Mann–Whitney U tests. Of the HIV-related factors, we considered the number of years lived with diagnosed HIV infection, CD4 cell count (<200, 200–500 and >500 cells/mm^3^), HIV viral load (undetectable (≤50 copies/mL or self-reported as undetectable), detectable), ART status (on ART, not on ART) and reported adherence to ART (took all ART drugs over the past 7 days, did not take all ART drugs/not on ART/not stated) at the time of the study visit.

### Analyses of factors associated with pain extent

Univariable and multivariable ordinal logistic regression was used to identify factors independently associated with the reported level of pain. For these analyses, individuals without information on menopausal status were excluded. Analyses were undertaken in a staged manner, allowing us to investigate the potential confounding or mediating effects of a range of factors, many of which are correlated: initially (*Stage 1*) models were fitted including only the demographic and lifestyle factors, with factors that were significant in these models (*p* < 0.05) being retained. In *Stage 2*, we considered whether the number of clinical comorbidities was additionally associated with pain after controlling for the factors identified in *Stage 1*. Finally, in *Stage 3*, we additionally considered the role of HIV-related parameters after controlling for the existing covariates in the model.

### Analyses of associations between pain level, depressive symptoms and insomnia symptoms

We also considered the association between reported pain level and severe depressive symptoms (PHQ4 ≥ 6) and insomnia symptoms (taking the approach used by Jean-Louis et al. 2012). For these analyses, we used multivariable logistic regression to describe the association between reported pain level and each outcome, adjusting for the factors identified as potential confounders in the Stage 3 model.

## Results

Of the 867 PRIME participants, 844 (97.4%) responded to the question about pain. There were no clinically meaningful or statistically significant differences between the 23 participants who did not complete this question and the remaining 844 participants regarding clinical site, ethnicity, year of completion, employment or educational status, having enough money to cover basic needs, menopausal status, age or depression score.

The women were predominantly of black African origin with most having been born outside the UK (Table 1). The median (interquartile range) age of the group was 49 (47, 53) years. Around two-thirds had completed A-levels or had a university degree/equivalent and just under half were working full-time. Around a fifth were pre-menopausal, 44.0% peri-menopausal and 35.1% post-menopausal. Only a minority reported current smoking, recent recreational drug use or harmful alcohol use.

**Table 1.** Demographic and lifestyle characteristics of study participants, overall and stratified by reported level of pain.

		All women	Level of pain			
			None	Moderate	Extreme	*p*-value
*n*		844	395	376	73	
Ethnic group, *n* (%)	Black African	593 (70.3)	276 (69.9)	269 (71.5)	48 (65.8)	
	White	102 (12.1)	51 (12.9)	45 (12.0)	6 (8.2)	
	Black other	74 (8.8)	33 (8.4)	31 (8.2)	10 (13.7)	
	Other/not stated	75 (8.9)	35 (8.9)	31 (8.2)	9 (12.3)	0.60
Age (years, *n* = 792)	Median (IQR)	49 (47, 53)	49 (47, 52)	50 (47, 53)	50 (48, 53)	0.11
Born in the UK, *n* (%) (*n* = 831)		123 (14.8)	60 (15.5)	56 (15.1)	7 (9.6)	0.41
Employment status, *n* (%)	Full-time	399 (49.1)	233 (59.0)	153 (40.7)	13 (17.8)	0.0001
Educational attainment*, *n* (%)	Low	324 (38.4)	139 (35.2)	151 (40.2)	34 (46.6)	
	High	520 (61.1)	256 (64.8)	225 (59.8)	39 (53.4)	0.12
Enough money to cover basic	All/most of the time	528 (62.6)	286 (72.4)	215 (57.2)	27 (37.0)	
needs, *n* (%)	Some/none of the time	316 (37.4)	109 (27.6)	161 (42.8)	46 (63.0)	0.0001
Menopausal status, *n* (%)	Pre-menopausal	173 (20.9)	103 (26.4)	59 (16.1)	11 (15.5)	
	Peri-menopausal	364 (44.0)	149 (38.2)	182 (49.6)	33 (46.5)	
	Post-menopausal	291 (35.1)	138 (35.4)	126 (34.3)	27 (38.0)	
	Not stated	16	5	9	2	0.002
Current smoker, *n* (%)		69 (8.4)	17 (4.4)	40 (10.9)	12 (16.4)	0.0002
Recent recreational drug use, *n* (%)		20 (2.4)	6 (1.6)	13 (3.5)	1 (1.4)	0.18
Harmful alcohol use, *n* (%)		69 (8.8)	29 (7.9)	36 (10.2)	4 (6.3)	0.41

*Educational attainment classified as Low (did not finish school, O-levels/GCSEs or equivalent, or not stated) or High (A-levels or equivalent, University degree or higher).

### Demographic and life-style associations with pain level

Of the 844 participants, 395 (46.8%) reported no pain, 376 (44.6%) moderate pain and the remaining 73 (8.7%) extreme pain. There were no significant differences between these three groups by year of survey, ethnic group, being UK-born or educational attainment (Table 1). However, as expected, women reporting extreme pain were less likely to be working full- or part-time (*p* = 0.0001) and were less likely to have enough money to cover their basic needs (*p* = 0.0001, Table 1). Whilst there was no significant difference in age between women in the three pain groups (*p* = 0.11), women reporting moderate or extreme pain were more likely to be peri- or post-menopausal than those reporting no pain (*p* = 0.002). The proportion of women reporting current smoking increased with pain level (*p* = 0.0002), but there were no differences in the proportions reporting recent recreational drug or harmful alcohol use.

In *Stage 1* of the multivariable logistic regression analyses, full-time work, having enough money to cover basic needs, current smoking and peri-/post-menopausal status were significantly associated with pain level and were retained for consideration in *Stage 2.* There was no evidence that the associations of pain with full-time work, having enough money to cover basic needs or current smoking differed by menopausal stage, with formal interaction tests all non-significant.

### Associations between clinical comorbidities and pain level

**Figure 1.**
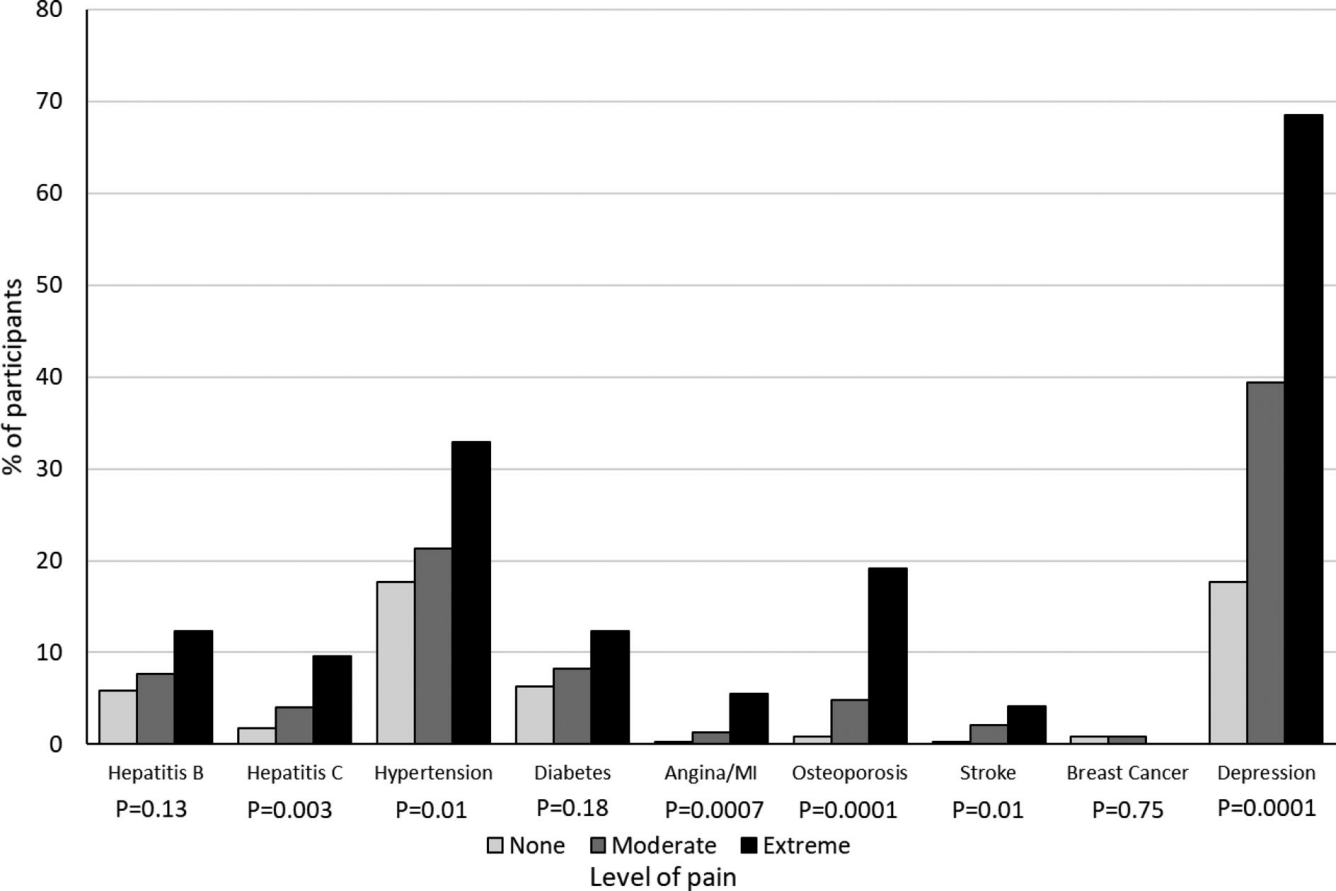
Frequency of diagnosed medical conditions, stratified by reported level of pain.

With the exception of breast cancer, which was reported by 6 women, all reported comorbidities increased in frequency as the pain level increased (Figure 1); the total number of reported comorbidities increased significantly from 0 (inter-quartile range 0–1) in pre-menopausal women, to 1 (0–1) and 2 (1–2) in women who were peri- or post-menopausal, respectively (*p* = 0.0001). In unadjusted analyses, the odds of more severe pain increased by 126% for each additional medical condition (OR 2.26 (95% CI 1.93, 2.66), *p* = 0.0001). After adjusting for full-time work, having enough money to cover basic needs, current smoking and peri-/post-menopausal status, each additional medical condition reported was associated with a doubling in the likelihood of more severe pain (adjusted OR 2.02 (1.70–2.38), *p* = 0.0001).

### Association between HIV parameters and pain level

Whilst women reporting moderate/extreme pain had been diagnosed with HIV for longer than those reporting no pain (*p* = 0.007), there were no strong associations with either nadir or latest CD4 count or HIV viral load (Table 2). Almost all women were currently on ART, with no difference between pain groups. ART adherence was less likely to be optimal in women reporting extreme pain (*p* = 0.01) and this trend was reflected in a lower proportion with an undetectable viral load, although this latter finding was not significant (*p* = 0.19). In a regression analyses, longer duration of diagnosed HIV infection remained significantly associated with pain level after adjustment for menopausal status only, and after adjustment for all previously identified confounders (unadjusted estimate (/additional year since diagnosis) 1.03 (1.01, 1.05), *p* = 0.002; adjustment for menopausal status 1.03 (1.01, 1.06), *p* = 0.001; adjustment for all confounders 1.02 (1.00, 1.04), *p* = 0.04). In contrast, whilst optimal adherence to ART was associated with pain extent in unadjusted analyses (0.57 (0.39, 0.84), *p* = 0.004) and after adjustment for menopausal status (0.57 (0.39, 0.84), *p* = 0.004), this effect was attenuated after additional adjustment for other potential confounders (0.78 (0.52, 1.17), *p* = 0.24).

**Table 2.** HIV-related parameters among study participants, overall and stratified by reported level of pain.

		All women	Level of pain			
			None	Moderate	Extreme	*p*-value
*N*		844	395	376	73	
Years diagnosed with HIV (*n* = 786)	Median (IQR)	14 (9, 18)	13 (9, 17)	14 (10, 19)	15 (10, 19)	0.007
Latest CD4 count (cells/mm^3^) (*n* = 745)	>500	509 (68.3)	249 (70.9)	222 (66.3)	38 (64.4)	
	200–500	187 (25.1)	79 (22.5)	90 (26.9)	18 (30.5)	
	<200	49 (6.6)	23 (6.6)	23 (6.9)	3 (95.1)	0.56
Latest viral load undetectable (*n* = 795)	*n* (%)	703 (88.4)	328 (88.2)	318 (90.1)	57 (81.4)	0.12
On ART (*n* = 817)	*n* (%)	798 (97.7)	372 (97.4)	355 (97.8)	71 (98.6)	0.80
Adherent to ART (*n* = 831)	*n* (%)	720 (86.6)	349 (90.0)	314 (84.6)	57 (79.2)	0.01

**Figure 2.**
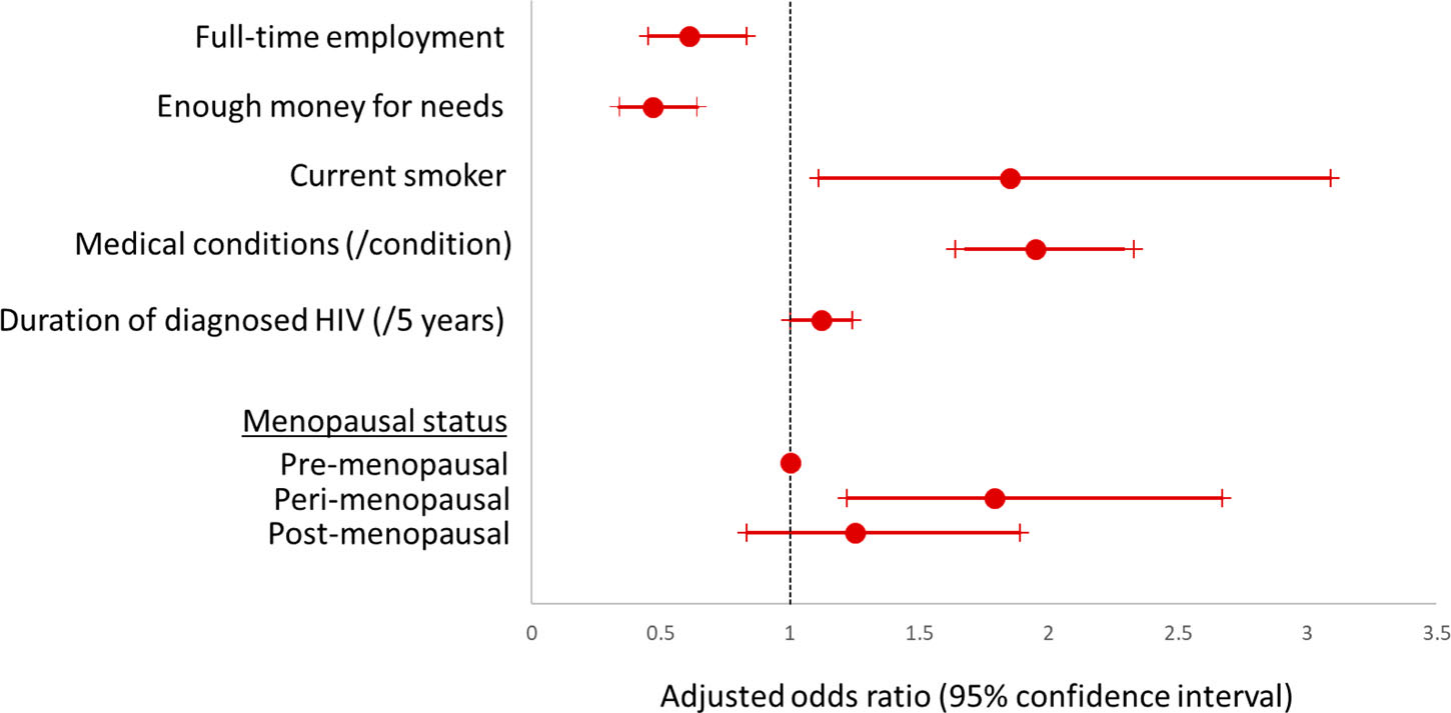
Final multivariable model for predictors of level of pain in women living with HIV.

The final multivariable model therefore included menopausal status, full-time employment, enough money for needs, current smoking, the total number of medical conditions reported and years of diagnosed HIV (Figure 2).

### Association between pain level and other menopause symptoms, and markers of physical and mental health

**Figure 3.**
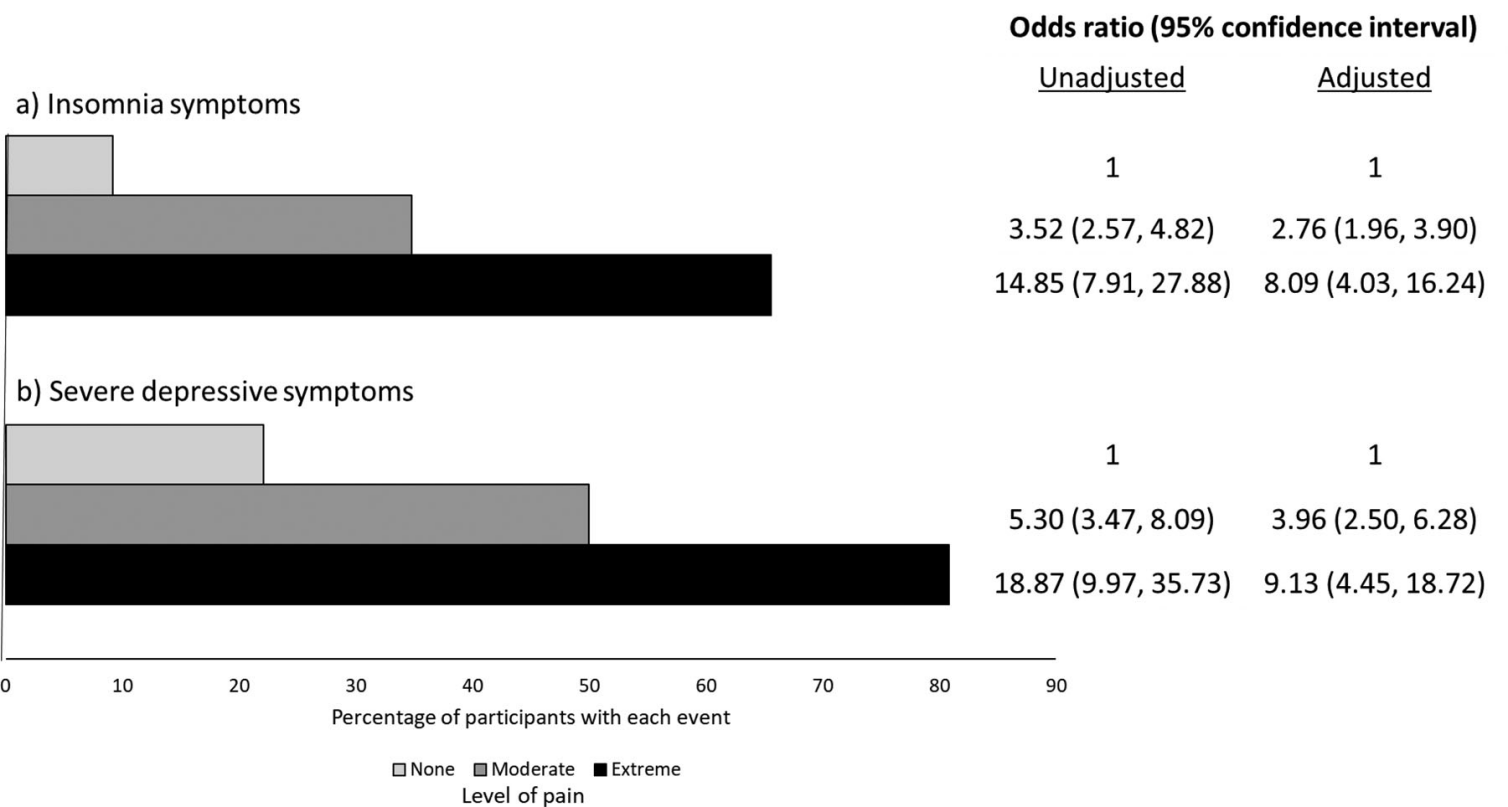
Prevalence of (a) insomnia symptoms and (b) severe depressive symptoms, stratified by reported level of pain, and results from unadjusted and adjusted logistic regression analyses of the associations between pain level and each outcome. Adjusted analyses include adjustment for full-time employment, enough money to cover basic needs, current smoking, the number of medical conditions, duration of diagnosed HIV and menopausal status.

Overall, 331/834 (39.7%) reported insomnia symptoms (80.8%, 50.0% and 22.1% in those with extreme, moderate and no pain, respectively, *p* = 0.0001) and 189 (25.1%) had severe depressive symptoms (65.6%, 34.8% and 9.2%, respectively, *p* = 0.0001) (Figure 3). Strong associations were seen between both moderate and extreme pain and insomnia symptoms both before (moderate pain: 3.52 (95% 2.57, 4.82); extreme pain: 14.85 (7.91, 27.88), *p* = 0.0001) and after (moderate pain: 2.76 (1.96, 3.90); extreme pain: 8.09 (4.03, 16.24), *p* = 0.0001) adjustment for potential confounders. Similarly, strong associations were also reported with severe depressive symptoms, both before (moderate pain: 5.30 (3.47, 8.09); extreme pain: 18.87 (9.97, 35.73), *p* = 0.0001) and after (moderate pain: 3.96 (2.50, 6.28); extreme pain: 9.13 (4.45, 18.72), *p* = 0.0001) adjustment for the same potential confounders.

## Discussion

In this large, nationally representative study of women living with HIV in the UK aged 45–60 years, we found that pain was commonly reported, was more extreme in peri- and post-menopausal women than in those who were pre-menopausal, and was associated with several markers of socioeconomic disadvantage and co-morbidity. We also report that increasing pain was strongly associated with severe depressive symptoms and sleep problems. Our findings highlight the importance of eliciting a history of pain and addressing symptoms in order to improve the wellbeing of women living with HIV.

Pain was one of the first symptoms to be reported among people with HIV, often being associated with treatment with the older so-called D-drugs, including didanosine, zalcitabine and stavudine (Stavros & Simpson, 2014), which are no longer commonly prescribed. However, even in the era of more modern ART regimens, pain remains common (da Silva et al., 2017; Merlin et al., 2012; Parker et al., 2014; Sabin et al., 2018). In the Pharmacokinetic Observations in PeoPle over fiftY (POPPY) Study (Sabin et al., 2018), the prevalence of reported aches and pains in the last month and current pain were both significantly higher in people with HIV over the age of 50 years (70.0%) than in either younger people with HIV (62.7%) or older HIV-negative controls (63.7%, *p* = 0.03), and was associated with more days off work or study, lack of full-time work, greater healthcare resource use and poorer mental health. Information about the prevalence of pain specifically among women with HIV, however, is more limited, with only a small proportion of POPPY participants being female. In an early analysis of 339 women participating in the Women’s Interagency HIV study (WIHS) (Richardson et al., 2009), over half had experienced pain in the past month. Whilst pain frequency and severity were not associated with age, the majority of women in the study were under the age of 40 years, and menopausal status was not considered. Pain was, however, associated with depressive symptoms.

Our findings suggest that pain in women living with HIV is more common in those with other comorbidities as well as in those who do not work full-time and those who lack enough money to cover basic needs. The finding of an association with comorbidities confirms previously reported associations in the general population (Larsson et al., 2019) as well as in those with HIV (Chukwurah et al., 2020). To restrict the length of study visit, only a limited number of age-related comorbidities, many of which have been reported to occur commonly in those with HIV, were studied. Unfortunately, we did not collect information on the treatment or management of these comorbidities, nor on other potential risk factors for these (e.g., Body mass index) and so are unable to rule out the fact that this association may be driven by confounding. Associations with lack of full-time employment and markers of low socio-economic status have also been reported (Dragioti et al., 2020; Ellis et al., 2020; Enel et al., 2019). Of note, the cross-sectional nature of our study prohibits us from determining the direction of any associations seen. For example, whilst women with chronic pain may find it difficult to participate in full-time work, anecdotally, many older migrant Black African women with HIV in the UK work in lower-paid jobs, despite a high educational attainment in their country of origin, and this in itself may result in an exacerbation of pain. Furthermore, the lack of distraction provided by full-time work may increase pain awareness. Longitudinal studies are essential in order to further elucidate the direction of these associations.

Pain is an established symptom of the menopause, but less is known about the experience of menopausal-related pain in the presence of HIV infection. Among >200,000 women veterans aged 45–64 years receiving care through the US Veterans Health Administration in 2014–2015 (Gibson et al., 2019), women with menopausal symptoms had nearly two-fold odds of chronic pain and multiple chronic pain diagnoses. Information specifically among women living with HIV, however, is scarce. In one study at a large outpatient HIV clinic in London, Sherr et al. (2016) found that 58.9%, 29.3% and 27.1% of women aged >45, respectively, reported low backaches, aching muscles and joints, and aches in the back of the neck or head. In a study of 975 men and 385 women with HIV in the US, pain burden scores were significantly higher in women than men (Schnall et al., 2018), although sleep problems did not appear to differ by sex. Among women, burden scores for pain and sleep were poorer in women who were amenorrhoeic compared to those who were menstruating, suggesting that the menopause (natural or surgical) was a risk factor. The biological pathways to the development of pain in people with HIV are complex, with the conceptual framework for chronic pain in those living with HIV (Merlin et al., 2014) bringing together mechanisms relating to biological (including HIV neuropathy osteonecrosis, psychiatric illness), psychological (including anger, fear, traumatic life events) and social factors (stigma, personal relationships and environmental stressors). Notably, however, menopausal status or hormonal factors do not feature explicitly within this model, and our findings suggest that this is a severe shortcoming.

Globally, around 50% of women aged 40–64 years report sleep disorders, predominantly insomnia (Makara-Studzinska et al., 2014), with the prevalence increasing in peri- and post-menopausal women. The association between pain and sleep disorders is established in the general population (Parish, 2009; Solomon et al., 2020; Terauchi et al., 2020). Multiple factors either precipitate or perpetuate the association between insomnia and the menopause (Proserpio et al., 2020), including pain. People with HIV may have many contributors to sleep problems, including obesity, respiratory problems, renal problems, depression, anxiety and diabetes, and several studies have reported a higher rate of sleep problems in people with HIV. In the POPPY-Sleep sub-study, 21.0% of people with HIV met criteria for insomnia, a rate that was over five times as high as that in similarly aged HIV-negative after adjustment for confounders (Kunisaki et al., 2020). Furthermore, those with widespread and regional pain consistently reported poorer sleep quality on all self-reported measures than those with no pain (Sabin et al., 2020). These associations were reduced, but remained significant, after adjustment for depressive symptoms.

As expected, we found a strong association between pain and depressive symptoms in our population of women with HIV. As with other associations, it is not clear whether pain drives negative mood, whether a negative mood enhances the experience of or attention to pain, or whether there is a complex mixture of both. Pain experience is linked to pain management, and those with low mood or depressive symptoms may lack the resources to cope with their pain. Our findings therefore have important implications for interventions and treatment, given the high global prevalence of depressive symptoms among women in this age group (Makara-Studzinska et al., 2014) and the complex bidirectional association between insomnia and depressive symptoms (Caruso et al., 2019).

Among women in the general population, there is a strong association between oestrogen deficiency and musculoskeletal pain, although robust evidence to support a causal association is lacking, as reviewed by Watt (2018). Although the link between pain, sleep disturbance and the menopause has been reported, information regarding the nature of this association and the potential role of HRT for women in the general population is scarce (Dias et al., 2019). However, one pilot study supports that resveratrol supplementation can reduce chronic pain and improve menopause-related quality of life in post-menopausal women (Thaung Zaw et al., 2020). Among women with HIV, the potential mechanisms are likely to be complicated by the additional role of inflammation due to HIV itself, and/or the use of ART drugs that are known to have an impact on sleep quality (Allavena et al., 2016). In a study of 25 men and 75 women living with HIV (Schnall et al., 2020), pain burden scores were highest in pre- and post-menopausal women, but surprisingly lower in peri-menopausal women. Muscle aches and pains were, however, associated with lower C-reactive protein (CRP) and interleukin (IL)-8 levels, and increased IL-6 levels, suggesting a role of inflammation in pain, with menstruation leading to inflammation. In a further small pilot study of 32 people with HIV, of whom 40% were women, muscle/joint pain was predicted by adiponectin, CRP and serum amyloid A (Zuniga et al., 2020).

Although we believe that our study is one of the largest studies specifically set-up to consider issues around the menopause in women with HIV, it does have some limitations. Our sample is representative of women living with HIV in the UK and, as such, participants in the study were predominantly of black African origin, and were virally suppressed on ART. However, this does mean that our findings may lack generalisability to populations of women of different ethnic origins and with a lower use or rate of viral suppression on ART. As noted earlier, our cross-sectional observational design limits our ability to determine the direction of associations and we cannot rule out the potential for unmeasured confounding or establish causality. We only had a single measure of pain, with no data on duration or extent of pain. Finally, whilst more detailed information was available on CD4 count and viral load at HIV diagnosis from linkage to patient records, this information was often missing and therefore we were unable to incorporate that into analyses.

In conclusion, pain is a common symptom among women with HIV and increases in prevalence as women experience the menopause. Given the strong association of pain with sleep and depressive disorders, there is a need to proactively address pain-related problems with women with HIV.
